# Utility of modified Laboratory Risk Indicator for Necrotizing Fasciitis (MLRINEC) score in distinguishing necrotizing from non-necrotizing soft tissue infections

**DOI:** 10.1186/s13017-021-00373-0

**Published:** 2021-05-26

**Authors:** Po-Han Wu, Kai-Hsiang Wu, Cheng-Ting Hsiao, Shu-Ruei Wu, Chia-Peng Chang

**Affiliations:** 1 Department of Emergency Medicine, Chang Gung Memorial Hospital, No.6, Sec. W., Jiapu Rd., Puzi City, Chiayi County 613 Taiwan (R.O.C.); 2 Department of Medicine, Chang Gung University, Taoyuan, Taiwan; 3 Department of Pediatrics, Kaohsiung Veterans General Hospital, Kaohsiung, Taiwan

**Keywords:** Necrotizing fasciitis, Modified LRINEC, Predictor

## Abstract

**Background:**

We conducted this study to promote a modified Laboratory Risk Indicator for Necrotizing Fasciitis (MLRINEC) score and evaluate the utility in distinguishing necrotizing fasciitis (NF) from other soft-tissue infections.

**Method:**

A retrospective cohort study of hospitalized patients with NF diagnosed by surgical finding was conducted in two tertiary hospital in southern Taiwan between January 2015 and January 2020. Another group was matched by controls with non-necrotizing soft tissue infections based on time, demographics, and immune status. Data such as infectious location, comorbidities, and laboratory findings were recorded and compared. Logistics regression were used to determine the association with NF after adjustment for confounders and MLRINEC score was developed by then. Receiver operating curve (ROC) and the area under the curve (AUC) were used to evaluate its discriminating ability.

**Result:**

A total of 303 patients were included; 101 in NF group and 202 in non-NF group. We added serum lactate and comorbid liver disease to the original LRINEC score and re-defined the cut-off values for 3 variables to develop the MLRINEC score. The cut-off value for MLRINEC score was 12 points with corresponding sensitivity of 91.8% and a specificity of 88.4%, and the area under ROC (AUC) was 0.893 (95% CI, 0.723 to 0.948; *p* < 0.01).

**Conclusion:**

MLRINEC score shows a high sensitivity and specificity in distinguishing NF from non-necrotizing soft-tissue infections. Patients with a MLRINEC score > 12 points should be highly suspected of presence of necrotizing fasciitis.

## Introduction

Necrotizing fasciitis (NF) is a severe, rapidly progressive disease that is characterized by the infection of subcutaneous tissue and fascia, resulting in extensive fascial necrosis [1]. The gold standard management for NF is rapid debridement and broad-spectrum antibiotics [2]. Even under rapid and timely management, the risk of mortality and morbidity, such as amputation and multiorgan dysfunction, remains high [3–5]. Early recognition of patients at risk of NF is an essential point to improve outcomes [6]. However, distinguishing necrotizing soft-tissue infections from non-necrotizing soft-tissue infections in their early course is difficult. Biochemistry laboratory markers, ultrasonography, and magnetic resonance imaging were used in early differential diagnosis [7, 8]. Wong et al. developed the Laboratory Risk Indicator for Necrotizing Fasciitis (LRINEC) score [9], which demonstrated high sensitivity in discriminating NF from other soft-tissue infections. However, recent studies on the LRINEC may have reported overpraised results. In different settings, the sensitivity of the LRINEC was 43.2–80.0% for a score of ≥ 6 and 28.6–68.4% for a score of ≥ 8 [10–12]. Furthermore, some studies have shown the LRINEC to be non-relevant [11, 13]. In this study, we modified the original LRINEC score based on the data on matched cases and controls to develop a new score, the modified LRINEC (MLRINEC) score, and this study was conducted to present the novel score and evaluate its discrimination ability.

## Material and methods

### Patient selection

Under the approval of institutional review board, a retrospective cohort study was conducted. The medical records of patients who met the inclusion criteria of surgically proven NF and who received management between January 2015 and January 2020 in two tertiary hospitals were reviewed. Selected comorbidities and initial laboratory values were extracted through medical chart review. In total, 101 patients with NF were identified and assigned to the case group. At the selected time window, control patients were randomly selected from 841 patients with an admission diagnosis of non-necrotizing soft-tissue infections, which was identified according to the International Classification of Diseases, Ninth Revision, Clinical Modification (ICD-9-CM) codes 528.3 (cellulitis and abscess of oral soft tissues), 681.00–681.9 (cellulitis and abscess of the finger and toe), and 682.0–682.9 (other cellulitis and abscess). NF cases were matched to the control patients in a ratio of 1:2 using the propensity score based on the following variables: age, sex, initial vital signs, admission time, and immune status (immunocompromised or not). The R software was used to perform the matching process using the “MatchIt” package, with a caliper value of 0.25 standard deviation of the logit of a propensity score. Later, 202 control patients were included in the control group.

### Data collection and measurement

Age, sex, vital signs in the emergency department (ED), admission date, the presence of comorbidities (i.e., cerebrovascular disease, heart disease, pulmonary disease, liver disease, kidney disease, peripheral vascular disease, malignancy, and diabetes), serum lactate, and LRINEC score, including C-reactive protein, total white blood cell count, hemoglobin level, blood glucose, sodium concentration, and serum creatinine, were analyzed. After data collection was completed, random chart reviews were performed to ensure accuracy. All blood samples were collected upon arrival to the ED.

### Statistical analysis and MLRINEC score

All data were analyzed using the statistical package for the social sciences software, version 20.0 (IBM Corp., Armonk, NY, USA). Continuous variables were analyzed using the *t* test, and categorical variables were analyzed using the chi-square test, except for cases where 20% of the cells had expected counts of less than 5, in which case, Fisher’s exact test was used. The receiver operating characteristic (ROC) curve to determine the optimal cut-off value for each variable was plotted, corresponding to the maximized Youden index. Area under the ROC curve (AUC) analysis was used to evaluate the discrimination ability of the MLRINEC score.

Logistic regression analysis was performed to determine the independent effects of each variable on the development of NF using the entry method after adjusting for age, sex, and initial vital signs. To establish the score system, odds ratio for independent variables was rounded up and the total score for each patient was calculated by summing up the scores (Table 2). Then, the ROC curve and AUC were used to indicate the discrimination ability of the MLRINEC score, and the optimal cutoff value and corresponding sensitivity and specificity were determined. *P* values of less than 0.05 were considered to be statistically significant.

## Results

In this study, 101 patients with NF were enrolled, and 202 patients with non-necrotizing soft-tissue infections were matched and included in the control group. Using the propensity score matching method, the patients in the NF and non-NF groups exhibited almost the same basic characteristics. The patients in the NF group had a higher incidence of liver and kidney diseases than those in the non-NF group (34.7% vs 14.9% for liver disease, *P* < 0.01 and 38.6% vs 12.4 % for kidney disease, *P* < 0.01). Elevated lactate levels were independently associated with the mortality rate in critically ill patients [14]. Even intermediate levels of initial serum lactate were an indicator of mortality, organ dysfunction, and shock in patients with severe sepsis in the ED [15]. In hospitalized patients, increased lactate levels indicated high mortality, mechanical ventilation, vasopressor requirement, and a high incidence of intensive care unit admission [16–18]. Due to the aforementioned reasons, we added serum lactate as a new variable in the MLRINEC score. All six original LRINEC score variables and serum lactate as a continuous or categorical variable were significantly different between the NF and non-NF groups (all *P* < 0.05) (Table 1).

**Table 1:** Clinical characteristics between NF and non-NF groups

Variable (n, % or mean ± SD)	NF group (***n*** = 101)	Non-NF group (***n*** = 202)	***P*** value
Age	57.1 ± 19.8	57.0 ± 19.5	0.91
Sex (male)	67 (66.3%)	134 (66.3%)	1.0
Systolic blood pressure	103 ± 18.3	111 ± 15.1	0.53
Heart rate	98 ± 13.5	102 ± 16.7	0.32
Body temperature	36.8 ± 1.9	36.5 ± 2.3	0.84
Malignancy	7 (6.9%)	12 (5.9%)	0.87
Heart disease	15 (14.9%)	27 (13.4%)	0.75
Pulmonary disease	11 (10.9%)	23 (11.4%)	0.59
Liver disease	35 (34.7%)	30 (14.9%)	< 0.01^*^
Kidney disease	39 (38.6%)	25 (12.4%)	< 0.01^*^
Peripheral vascular disease	9 (8.9%)	12 (5.9%)	0.03^*^
Diabetes mellitus	36 (35.6%)	21 (10.4%)	< 0.01^*^
Hypertension	15 (14.9%)	28 (13.9%)	0.18
Immunosuppressants use	6 (5.9%)	10 (5.0%)	0.94
CRP	75.6 ± 21.6	29.4 ± 19.7	< 0.01^*^
CRP > 30 (mg/dL)	58 (57.4%)	49 (24.3%)	< 0.01^*^
WBC	16.2 ± 6.4	11.1 ± 3.6	0.01^*^
WBC > 15 (× 10^4^ /uL)	41 (40.6%)	51 (25.2%)	< 0.01^*^
Hemoglobin	10.5 ± 3.8	12.1 ± 2.9	0.04^*^
Hemoglobin < 11(g/dL)	38 (37.6%)	48 (23.8%)	0.02^*^
Blood glucose	168 ± 47.1	132 ± 35.8	0.02^*^
Blood glucose ≧ 180(mg/dL)	29 (28.7%)	25(12.4%)	< 0.01^*^
Sodium	135 ± 4.5	139 ± 3.6	0.03^*^
Sodium < 135 (mEq/L)	28 (27.7%)	24 (11.9%)	0.01^*^
Lactate	18.3 ± 6.5	10.6 ± 4.8	< 0.01^*^
Lactate > 18 (mg/dL)	32 (31.7%)	22 (10.9%)	< 0.01^*^
Creatinine	1.6 ± 0.7	1.2 ± 0.4	0.02^*^
Creatinine ≧ 1.6 (mg/dL)	31 (30.7%)	24 (11.9%)	< 0.01^*^

*CRP* C-reactive protein, *WBC* white blood cell

*P < 0.05

**Fig. 1:**
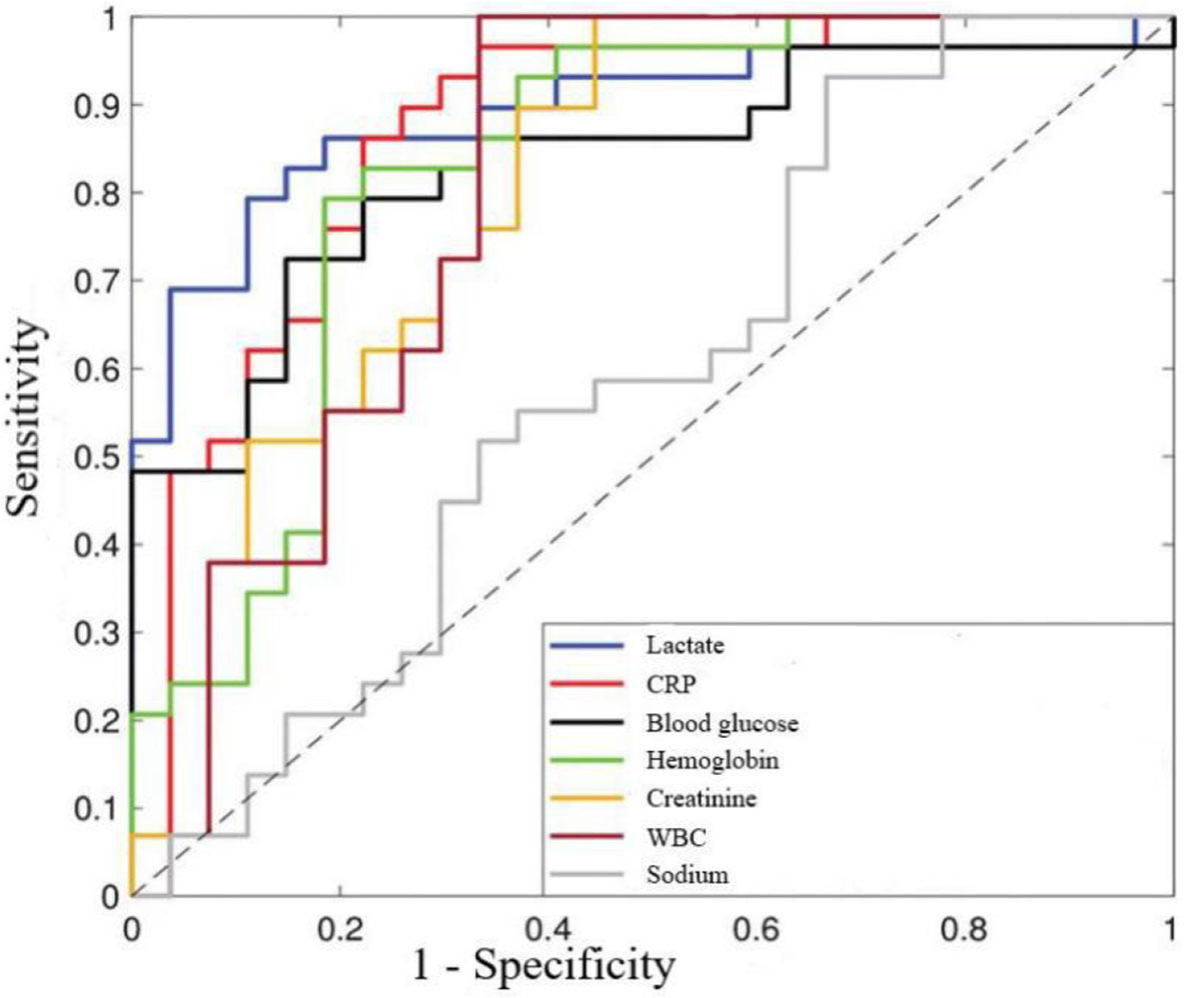
The ROC and AUC for the CRP, WBC, blood glucose, serum lactate creatinine, sodium and hemoglobin. Their respective AUC from 0.667 (95% CI, 0.591 to 0.827) to 0.898 (95% CI, 0.842 to 0.961)

Figure 1 shows the ROCs for seven variables, which exhibited the discriminating ability of the MLRINEC score, with an AUC ranging from 0.667 (95% confidence interval (CI), 0.591–0.827) for sodium to 0.898 (95% CI, 0.842–0.961) for serum lactate level (all *P* < 0.05). The optimal cutoff values were 30 mg/dL for C-reactive protein (CRP), 15 × 10^4^/uL for white blood cell (WBC) count, 180 mg/dL for blood glucose, 11 g/dL for hemoglobin, and 135 mEq/L for sodium. Their corresponding sensitivity was low to high, from 0.498 for creatinine level to 0.893 for lactate, whereas their specificity was moderate to high, from 0.554 for sodium to 0.912 for serum creatinine (Table 2).

**Table 2:** The optimal cut-off value, AUC, and ROC for calculated variables

Variable	AUC	95% CI Lower limit	95% CI Upper limit	*P* value	Cut-off value	Sensitivity	Specificity
CRP	0.863	0.803	0.927	< 0.01^*^	30 mg/dL	0.861	0.873
WBC	0.691	0.598	0.792	0.02^*^	15 × 10^4^ /uL	0.528	0.916
Blood glucose	0.765	0.671	0.856	< 0.01^*^	180 mg/dL	0.692	0.758
Creatinine	0.728	0.639	0.823	< 0.01^*^	1.6 mg/dL	0.498	0.951
Hb	0.734	0.645	0.815	0.01^*^	11 g/dL	0.682	0.765
Sodium	0.667	0.591	0.827	0.02^*^	135 mEq/L	0.883	0.554
Lactate	0.898	0.842	0.961	< 0.01^*^	18 mg/dL	0.893	0.885

*CI* confidence interval, *CRP* C-reactive protein, *WBC* white blood cell, *Hb* hemoglobin

*P < 0.05

**Fig. 2:**
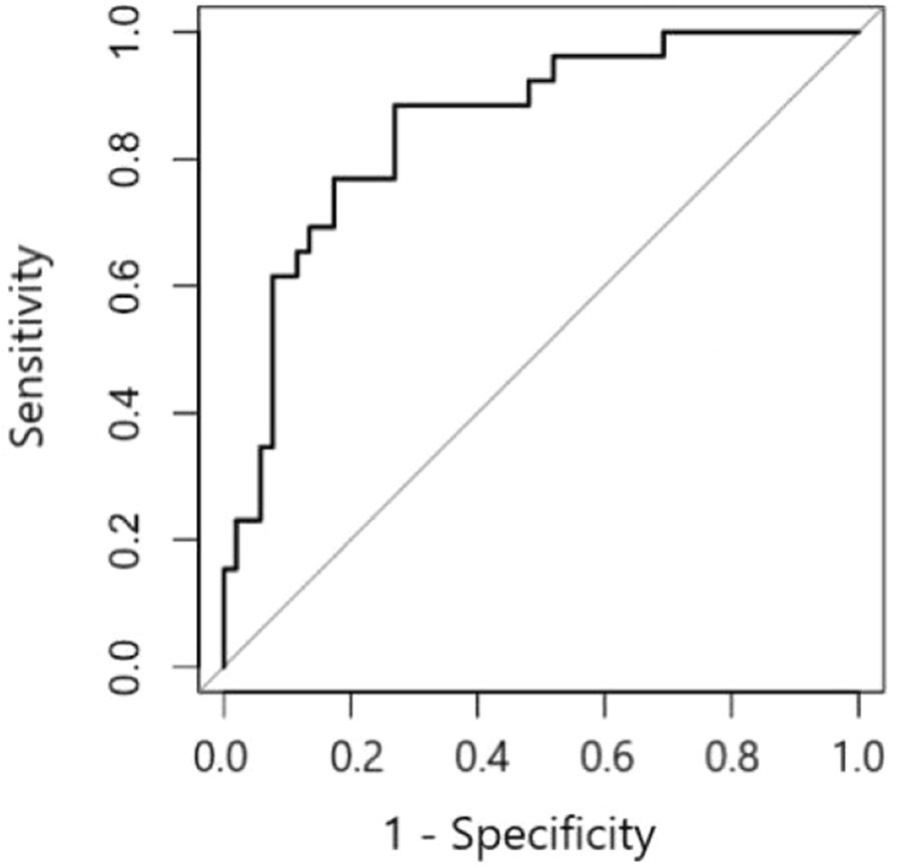
This figure shows the ROC for the modified LRINEC, sensitivity of 91.8%, and specificity of 88.4%, corresponding to an optimal cut-off of 12 points and the AUC of 0.893

Table 3 describes the association of the variables with NF, based on which the corresponding score was assigned. CRP > 30 mg/dL and serum lactate > 18 mg/dL were assigned the highest score of 4 points; serum creatinine ≥ 1.6 mg/dL and comorbid liver disease were assigned the score of 3 points; and WBC count > 15 × 10^4^/uL, hemoglobin < 11 g/dL, and blood glucose ≥ 180 mg/dL were assigned the score of 2 points. Sodium levels of < 135 mEq/L were assigned 1 point. We estimated the total score for each patient based on the assigned score of eight variables and constructed the combined ROC. The results showed that the MLRINEC score had a sensitivity of 91.8% and a specificity of 88.4%, and the AUC was 0.893 (95% CI, 0.723–0.948; *P* < 0.01) (Fig. 2).

**Table 3:** Assigned score for variables based on association with NF

Variables	Association magnitude with NF		*P* value	Assigned score
	OR	95% CI		
CRP > 30 (mg/dL)	4.32	1.95–9.18	< 0.01^*^	4
WBC > 15 (× 10^4^/uL)	2.38	1.27–6.37	0.01^*^	2
Hemoglobin < 11(g/dL)	2.14	2.01–9.53	0.02^*^	2
Blood glucose≧ 180(mg/dL)	2.48	1.36–5.12	< 0.01^*^	2
Sodium < 135 (mEq/L)	1.38	1.04–5.88	0.02^*^	1
Creatinine≧ 1.6 (mg/dL)	2.85	1.98–7.55	0.01^*^	3
Lactate > 18 (mg/dL)	4.49	2.66–12.54	< 0.01^*^	4
Liver disease	2.76	1.58–6.49	< 0.01^*^	3

*CI* confidence interval, *CRP* C-reactive protein, *WBC* white blood cell, *Hb* hemoglobin, *NF* necrotizing fasciitis, *OR* odds ratio

*P < 0.05

## Discussion

NF should be treated early and aggressively, and a prompt surgical intervention can prevent high-risk factors that lead to mortality. A prediction model based on laboratory markers may be useful in its early stage. Based on the original LRINEC score proposed by Wong et al. [9], we made some modifications to optimize the original LRINEC score to the MLRINEC score. The results showed that the MLRINEC score could discriminate NF from other soft-tissue infections, with high sensitivity (91.8%) and specificity (88.4%) when the cut-off value was determined to be 12 points, corresponding to the AUC of 0.893 (95% CI, 0.723–0.948; *P* < 0.01).

Because NF and its rapidly progressive infection remain associated with high mortality, the LRINEC score, developed by Wong et al. [9] based on readily available laboratory markers, has been consistently evaluated for its efficacy in various studies. Variable sensitivity ranging from 28.6 to 88.5% [10, 19–22] was found. These results may be associated with race, ethics, demographics, bacterial species, and timing of blood sampling for laboratory tests. Besides, some well-established comorbidities associated with infectious diseases, such as diabetes and kidney disease, and the use of immunosuppressants are not included in the original LRINEC score. In the MLRINEC score, we made several modifications to the original LRINEC score. Firstly, we added liver disease, which was significantly more prevalent in patients with NF than in patients without NF and it was also independently associated with NF. Besides, we added serum lactate level, which was confirmed to be associated with critical conditions and NF mortality [23]. Second, we redefined the cut-off values for CRP, total WBC count, and hemoglobin level to be 30 mg/dL, 15 × 10^4^/uL, and 11 g/dL, respectively. At such cutoff values, each variable could discriminate NF from other soft-tissue infections, and each variable was identified to be independently associated with NF after adjusting for confounders.

The clinical value of the MLRINEC score was determined by its sensitivity and specificity. The MLRINEC score could stratify patients into high- and low-risk categories for NF and help make critical decisions for duty surgeons. For high-risk patients, serial MLRINEC score monitoring may be useful for stopping the progression of NF. An early and aggressive surgical intervention may reduce mortality and related complications in high-risk patients. In the MLRINEC score, we dichotomized the laboratory variables but did not consider extreme cases, such as leukopenia, sepsis, and hematologic malignancy [24, 25]. These should alert physicians of the possibility of the presence of life-threatening conditions.

This study still has some limitations. First, this study lacks external validation. The validity of the MLRINEC score still requires other studies to confirm. Second, the retrospective design of this study had its inherent limitation in data collection, such as unverified comorbidities. Third, the sample size is relatively small due to the rarity of NF. Fourth, this study aimed to develop a modified score based on the LRINEC, so we did not include other possibly closely related inflammatory/immune variables, such as serum albumin and blood coagulation factors.

## Conclusions

In summary, we developed the MLRINEC score using a retrospective design. The risk stratification score demonstrated high sensitivity and specificity in the differential diagnosis of NF and other soft-tissue infectious and may be useful in providing necessary information for reasonable suspicion of NF. The validity of the MLRINEC score still needs to be confirmed by further studies.

## Data Availability

All the data will be available upon motivated request to the corresponding author of the present paper.

## Abbreviations

AUC: Area under curve
CI: Confidence interval
CRP: C-reactive protein
ED: Emergency department
Hb: Hemoglobin
ICD-9-CM: International Classification of Diseases, Ninth Revision, Clinical Modification
LRINEC: Laboratory Risk Indicator for Necrotizing Fasciitis
MRI: Magnetic resonance imaging
NF: Necrotizing fasciitis
OR: Odds ratio
ROC: Receiver-operating characteristics
SD: Standard deviation
WBC: White blood cell
