# Correction: Utility of modified Laboratory Risk Indicator for Necrotizing Fasciitis (MLRINEC) score in distinguishing necrotizing from non-necrotizing soft tissue infections

**DOI:** 10.1186/s13017-022-00450-y

**Published:** 2022-09-17

**Authors:** Po-Han Wu, Kai-Hsiang Wu, Cheng-Ting Hsiao, Shu-Ruei Wu, Chia-Peng Chang

**Affiliations:** 1 Department of Emergency Medicine, Chang Gung Memorial Hospital, No. 6, Sec. W., Jiapu Rd., Puzi City, Chiayi County, 613 Taiwan (R.O.C.); 2 Department of Medicine, Chang Gung University, Taoyuan, Taiwan; 3 Department of Pediatrics, Kaohsiung Veterans General Hospital, Kaohsiung, Taiwan

## Correction: World Journal of Emergency Surgery (2021) 16:26 10.1186/s13017-021-00373-0

Following the publication of the original article [1], the text under "Ethics approval and consent to participate" has to be changed

from

The institutional review board of Chia-yi Chang Gung Memorial Hospital approved this retrospective study (100-4178B) and (201900447B0C601). Consent to participate was not applicable.

to

The institutional review board of Chia-yi Chang Gung Memorial Hospital (IRB number: 100-4178B0C504 and 201900447B0C601) and Kaohsiung Veterans General Hospital (IRB number: VGHKS18-CT6-04) were approved this retrospective study. Consent to participate was not applicable.

The original article has been corrected.
